# The use of creative and humorous designs as vehicles for health education and infection control

**DOI:** 10.1186/2047-2994-4-S1-I12

**Published:** 2015-06-16

**Authors:** SWL Soh

**Affiliations:** 1 K Ventures, Singapore

## Introduction

ilovemicrobes.com^TM^ was started in late 2014 by a microbiologist cum ex-lecturer at a Singapore institute of higher learning. The concept was to incorporate humour and creative designs to give a glimpse of the world of microbes and their impacts in human health and disease.

## Objectives

The concept was to incorporate humour and creative designs to give a glimpse of the world of microbes and their impacts in human health and disease.

## Methods

Each design is scientifically-based on microscopic or macroscopic attributes of the real image of the actual microbes magnified up thousands if not millions times over.

The uniqueness of these microbes’ designs are not only to generate curiosity but also to demystify the diseases caused in a fun and approachable manner.

Microbes are known to cause disease. However, there is still a sense of complacency and resignations when it comes to infectious disease. The failure of people to exercise appropriate precaution and infection control processes are most likely due to ignorance and the lack of awareness.

Thus, the importance of a vehicle, which can capture the attention of people, especially children to be aware of the existence of these infectious agents, and to understand the way they caused infections; and finally as to how we can performance interventions to reduce the risk of infections are of significance.

## Results

For viewing of the characters designed, please go to http://www.ilovemicrobes.com

## Conclusion

It is the vision of ilovemicrobes.com^TM^ to promote Health Education and Infection Control in a fun and exciting way!

## Disclosure of interest

None declared.

